# Participatory Design and Process Testing to Optimize Utility, Usability, and Acceptability of a Mobile Game for Promoting Evidence-Driven Public Health Decision-Making in Resource-Constrained Settings

**DOI:** 10.3389/fdgth.2021.788557

**Published:** 2022-01-04

**Authors:** James Douglas Sinnatwah, Hajah Kenneh, Alvan A. Coker, Wahdae-Mai Harmon-Gray, Joelyn Zankah, Liam Day, Emma Hubbell, Michael J. Murphy, Mandy Izzo, David Kong, Peter Sylwester, Qinghua Long, Elena Bertozzi, Laura A. Skrip

**Affiliations:** ^1^School of Public Health, College of Health Sciences, University of Liberia, Monrovia, Liberia; ^2^Ministry of Health, Monrovia, Liberia; ^3^Department of Game Design and Development, Quinnipiac University, Hamden, CT, United States; ^4^Institute for Disease Modeling, Global Health, Bill and Melinda Gates Foundation, Seattle, WA, United States

**Keywords:** participatory design processes, evidence-based decision making, Liberia, research utilization, public health frontlines, mobile game-based learning

## Abstract

Innovative game-based training methods that leverage the ubiquity of cellphones and familiarity with phone-based interfaces have the potential to transform the training of public health practitioners in low-income countries such as Liberia. This article describes the design, development, and testing of a prototype of the *Figure It Out* mobile game. The prototype game uses a disease outbreak scenario to promote evidence-based decision-making in determining the causative agent and prescribing intervention measures to minimize epidemiological and logistical burdens in resource-limited settings. An initial prototype of the game developed by the US team was playtested and evaluated by focus groups with 20 University of Liberia Masters of Public Health (UL MPH) students. Results demonstrate that the learning objectives—improving search skills for identifying scientific evidence and considering evidence before decision-making during a public health emergency—were considered relevant and important in a setting that has repeatedly and recently experienced severe threats to public health. However, some of the game mechanics that were thought to enhance engagement such as trial-and-error and choose-your-own-path gameplay, were perceived by the target audience as distracting or too time-consuming, particularly in the context of a realistic emergency scenario. Gameplay metrics that mimicked real-world situations around lives lost, money spent, and time constraints during public health outbreaks were identified as relatable and necessary considerations. Our findings reflect cultural differences between the game development team and end users that have emphasized the need for end users to have an integral part of the design team; this formative evaluation has critically informed next steps in the iterative development process. Our multidisciplinary, cross-cultural and cross-national design team will be guided by Liberia-based public health students and faculty, as well as community members who represent our end user population in terms of experience and needs. These stakeholders will make key decisions regarding game objectives and mechanics, to be vetted and implemented by game design experts, epidemiologists, and software developers.

## Introduction

Mobile game-based learning (mGBL) has been increasingly applied to enhance learning motivation in a technology-driven world (1–3). Mobile game-based learning enables educators to connect the classroom and the real world by introducing skills and content as students engage with authentic scenarios and settings on their phones or tablets (4). In recent years, greater access to mobile technology has extended not only the potential reach of mGBL, but also user willingness and eagerness to engage with technology-based learning platforms in resource-constrained settings (5, 6). Of note, particularly dramatic increases in mobile phone penetration have been observed in sub-Saharan Africa (SSA), with an estimated 747 million SIM connections covering 75% of the population and more than half of adults owning a smartphone (7).

In resource-constrained settings, efforts have been made to develop learning tools that require minimal memory and bandwidth to account for connection and technology constraints and that draw from local context and culture to increase motivation and engagement in education (8, 9). For example, the multiplayer BaghLearn mobile game incorporates elements from Bagh-Chal, a popular board game in rural Nepal, with an algorithm designed to teach computational education (10). Likewise, a mobile-based Yoruba Language Tutor was developed to encourage and support learning of the Yoruba language among young Nigerians to reduce a growing divide between generations and the loss of indigenous languages (11). Ongoing development of context-specific mGBL can be used to address critical skills and knowledge gaps, from poor performance on regional, standardized math tests (12) to the lack of evidence-based decision-making in approaches to addressing ongoing public health threats (13).

In sub-Saharan Africa, the emergence—and re-emergence—of public health threats, such as Ebola, COVID-19 and Lassa fever, expose the vulnerabilities in surveillance efforts and health systems. The absence of rapid detection and adequately informed and resourced control measures repeatedly leads to the systems becoming severely overwhelmed (14–16). There is an urgent need to recognize barriers and harness opportunities for improvement, as limited in-country capacity to make timely decisions and implement evidence-based interventions hinders early detection and prevention of widespread morbidity and mortality. Gaps exist not only in the availability of context-specific evidence but also in the understanding of how to utilize research evidence (17). These challenges include: limitations in demand for evidence, capacity to use evidence, open access to data, time or opportunity to do research, and trust in the quality of evidence produced (17–20). While such challenges are widespread and persistent, recent, increased investment in research capacity-building has highlighted opportunities to harness growing interest in research and to generate more context-specific research evidence (21, 22).

Heightened interest around research capacity building coupled with accessibility to cell phones is creating an opportunity to use mGBL to aid in promoting more evidence-based decision-making by individuals serving at the public health frontlines. Here we describe a qualitative study investigating perceived utility and usability of a prototype mobile application that was designed to encourage public health practitioners to use quantitative research evidence to inform decisions for effective and efficient problem-solving. Three focus group discussions were conducted with a targeted sample of app users to gather their opinions on the content and features of the prototype considering their levels of experience with public health research and practice as well as their levels of experience with mGBL approaches. This paper presents key findings about users' perceptions of the app and its proposed learning objectives as well as lessons learned to inform the ongoing, intercultural, cross-national design process.

## Materials and Methods

### Overview of Prototype

The *Figure It Out* mobile application is envisioned to motivate the use of findings from quantitative research in decision-making at the frontline. The approach involves use of “real-world” scenarios as case studies to put users in the roles of key public health personnel, such as surveillance officers, Ministry of Health leadership, and community health workers. The game mechanic empowers players to make a series of decisions to determine the cause of a lethal outbreak at the lowest “cost” of time, money, and human life.

The prototype of the app includes a single scenario that is based on a meningococcal disease outbreak in Liberia (23). Users take on the role of a surveillance officer and are confronted with the situation of an unexplained cluster of deaths among wedding attendees. They must make decisions about actions for collecting information and determining the causative agent, with consideration of resource constraints. In the process, two minigames are introduced to guide users through the process of conducting an internet search with terms gleaned from the case investigation. After the causative agent is confirmed, users are presented with short quantitative results summaries from three scientific studies to guide their decision-making around how to intervene. A process of elimination approach is used. At each step, users can make a less favorable decision that results in money, time, and/or additional lives lost. Feedback is given to get users back on track, but they do not recover lost resources. Completing the process without running out of time or money constitutes a win. At each decision-making point–importantly, both before and after evidence has been searched for–users are asked to slide a “Confidence Bar” to indicate how confident they were in their decisions. This tool and outcome measurement were being tested as a potential metric for assessing the perceived utility of research evidence in decision-making (i.e., by enhancing confidence in decisions made once they were based on evidence) in anticipation of an outcome evaluation.

The prototype scenario was developed in Twine (24), with two linked minigames developed in Unity® WebGL (25). The prototype is available at http://ardeaarts.org/IDM/figureItOut.html.

### Design Process to Date

The *Figure It Out* prototype was developed by a multidisciplinary team of educators, software developers, game designers, and epidemiologists from institutions in Seattle, US, Connecticut, US, and Monrovia, Liberia. The complementary strengths of team members have facilitated effective collaboration, despite geographic distance and disparate time zones. The initial idea for *Figure It Out* was generated based on experience living and working in Liberia during and after the 2014–2015 Ebola outbreak. Initial iterations of the idea were prepared in Adobe XD by a team of developers and scientists at the Institute for Disease Modeling (IDM). Engagement with faculty and students in Game Design & Development at Quinnipiac University (QU) led to refinement of game mechanics and player feedback. In anticipation of a completed game launch using an Android phone app, testable prototypes were developed using Twine and Unity for the interactive minigames. Ahead of focus group testing, four research assistants were recruited from the University of Liberia School of Public Health (UL SOPH) Masters of Public Health (MPH) program. The students navigated through the prototype and offered feedback that was incorporated into the final version used in the focus group testing. The students were also responsible for recruitment efforts and were trained in facilitating focus groups and transcribing audio-recorded feedback.

### Description of Study Sample

In April 2021, three focus groups were conducted with a total of 20 MPH students at the UL SOPH. To be eligible for the study, participants had to be current students at the UL SOPH MPH program, be at least 18 years old, and provide consent on the IRB-approved form. The student body at the UL SOPH MPH program totals 120 individuals and reflects a broad range of public health experience, from little professional exposure to decades at the frontlines. This student population represents the actual end user audience for the app and was therefore appropriate and convenient to sample from at this formative phase in the research. Students with professional experience in the public health sector were specifically targeted with recruitment efforts, although all MPH students were eligible to participate. A recruitment email with a sign-up link for three predetermined dates/times was sent to the student body. The four research assistants also verbally disseminated information about the focus groups in their classes.

Individual focus groups were not defined by particular characteristics as participation was open to MPH students representing the three concentrations (i.e., Applied Epidemiology, Health Systems Management, and Environmental Health) offered at the program and the two cohorts of currently enrolled students (i.e., first-year and second-year students). An email was shared with all students in the University of Liberia MPH program for them to select the most convenient date and time from three options, if they were interested in participating. The three date/time options were selected by the research team to be in the late afternoon hours and to not conflict with any MPH courses, so as to minimize work- and school-related barriers to participation. A maximum number of sign-ups per focus group was included to ensure balance in numbers of participants per group. Groups derived from the process were expected to be comparable and representative of the student population, although no formal data collection on participants sociodemographic or school-related characteristics was conducted at this phase of the project. Furthermore, groups were smaller in number (six or seven persons), as has been recommended for ease of management of the group and for ensuring that there is synergy to reduce the opportunity for one or two voices to dominate the group session (26).

The total sample size of 20 participants across three focus groups was feasible given resource constraints around the project, was expected to provide representative feedback from the UL SOPH MPH student body, and was consistent with other qualitative studies in low-resource settings (27–29).

### Focus Group Testing

Resource constraints in the focus groups made it impossible to test the prototype using individual playthroughs on personal devices. Specifically, among target users in Liberia, logistical and technical constraints around personal access to laptops and downloading new software, such as a prototype API, would have taken significant time and human resources to facilitate individual-level access to the prototype game. The design team took such constraints into consideration and decided to use a web-based version of the game for the prototype to minimize time for troubleshooting technological challenges and maximize time for feedback during the study period. Ultimately, the design team will develop the game for use on mobile phones, which are generally more ubiquitous than personal laptops and which will allow for offline play after initial download, and will develop a plan for assisting target users with downloading questions and issues that fell out of the purview of this evaluation phase.

Focus groups were shown the prototype on a projector with research assistants soliciting actions from the group at decision points and then implementing them in the prototype. Feedback from participants was solicited using semi-structured focus group guides. The four student research assistants served as facilitators. They initially walked through the prototype and asked participants to make recommendations at decision points in the game. After key stopping points, the facilitators asked questions about clarity of the game, engagement, and purposefulness. These indicators are consistent with those examined in similar studies (30–33). The sessions were recorded; although both audio and video recordings were intended, electricity and technology challenges prevented consistent video recordings. During this round of testing, individual questionnaires were not used since participants did not individually play through the prototype. Future evaluations will ensure adequate time and support for facilitating playthroughs and individual-level data collection.

Audio transcripts from the three focus groups were assessed using thematic analysis. Thematic analysis for qualitative research has been widely used for formative, introductory research to guide future evaluations and is recognized as an approach for providing trustworthy and valuable results when conducted in a robust way (34, 35). The study was reviewed and approved by the University of Liberia-Pacific Institute for Research and Evaluation Institutional Review Board. Written informed consent was obtained from each participant and identifiers were used instead of participant names in both the audio recording and transcripts to ensure confidentiality.

## Results

The focus groups offered insight into target users' learning interests, learning styles, and exposure to mobile applications. Full transcripts are available in the Supplementary Material. Here we highlight key thematic areas that emerged from the discussions during a group-level, guided playthrough of the prototype.

### Theme 1: Learning Needs Were Addressed

**Figure 1:**
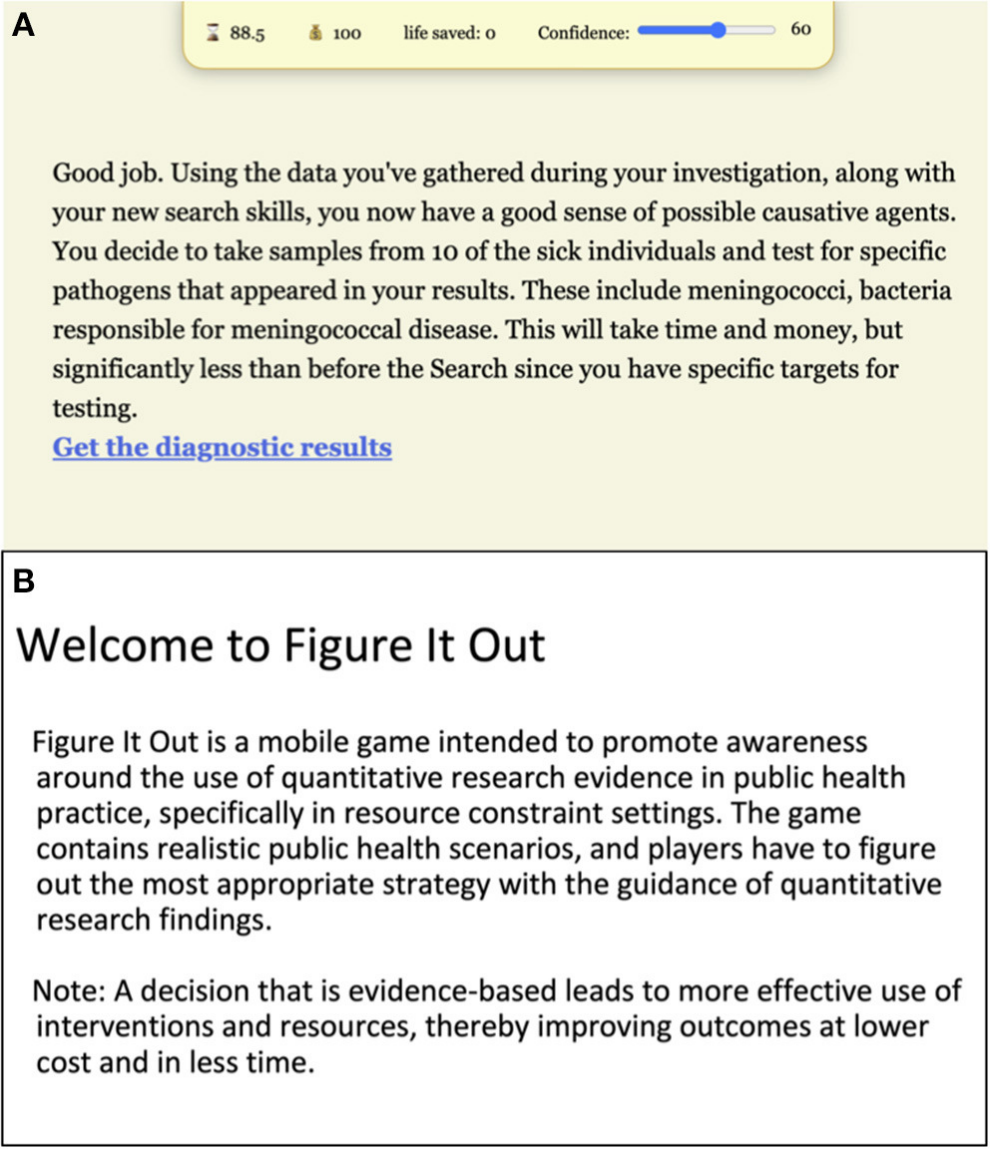
Feedback emphasizing learning goals in the prototype. Players encounter feedback screens **(A)** that emphasize how identifying and/or utilizing evidence can improve public health outcomes, even with fewer resources used in the process. To ensure that this goal is emphasized explicitly and upfront, an introductory screen will be introduced **(B)** at the start of the scenario during the next iteration of the scenario.

Participants indicated that they had learned as a result of interacting with the prototype. There was general agreement that the *Figure It Out app* is a good idea and will provide knowledge and guidance around identifying and utilizing public health research in the decision-making process (Figure 1A). The ability to make more informed decisions has implications for time and money spent in public health emergencies, as well as lives lost.

“*The app is good and I learned a lot from the app. One of those things learnt is that when an investigation is ongoing in the field, the best thing is to research before making decision.” (Participant 2, Focus Group 1)*

“*I've understood that when an investigation is ongoing in the field, there's a need to go deeper in your search before making decision.” (Participant 2, Focus Group 1)*.

“*I learned from the scenario that whenever there is an outbreak, you have limited resources (time and money) to make decision. The best way to approach is to research and come out with the best possible decision/recommendation for action, rather than taking immediate action (without evidence) and spending more money on issue that could cost less if action taken was based on evidence.” (Participant 3, Focus Group 1)*

“*The most interesting part of the game is the decision making process. It is clearly stated that choosing any of the recommendations could either lead to saving lives or losing lives.” (Participant 5, Focus Group 1)*

“*Figure it out is... using the app, you will not just rush in making decision you must think critically. This app…, exactly, I will recommend it to others.” (Participant 4, Focus Group 3)*

### Theme 2: Approaches to Problem-Solving and Decision-Making

The scenario in the prototype game is intended to provide skills that can be applied across public health decision-making scenarios. However, this warrants an understanding of how target players typically approach problem-solving and decision-making. Feedback indicated that players may desire specific skills that are applicable to specific problems rather than general skills that can be applied across problems.

“*...How will the app, take for an example I'm in Nimba, and there's an incident in Nimba. How will the app just understand that this incident has occurred and something needs to be done?” (Participant 4, Focus Group 3)*

“*I think there's a set standard procedure in case of investigating an outbreak. I think the app should follow that path, that pathway. So, if we are to play the game to get more information, the app should not confuse us to do something that is not really necessary.” (Participant 1, Focus Group 2)*

Feedback revealed that problem-solving strategies preferred by the target players are direct and unidirectional, rather than iterative and through trial and error.

“*The app is great, but the aspect of giving options so that one of the actions (test guests, interview guest and test environment) to be taken is not that clear. The game should not have options while in the field because you want to make real time decision, not to play puzzles. The app should instruct you on what to do and what not to do.” (Participant 6, Focus Group 1)*

“*The game should be straight and to the point. It shouldn't be giving options that are not needed at a particular time.” (Participant 3, Focus Group 3)*

‘*I will like this app to be something that people will just go straight and get results instead of just going. It should be concise instead of it just beating around the bush.... The reason for this app is to investigate, so going back and forth is very, very much time consuming.” (Participant 3, Focus Group 3)*

### Theme 3: Challenges to Navigating the App

**Figure 2:**
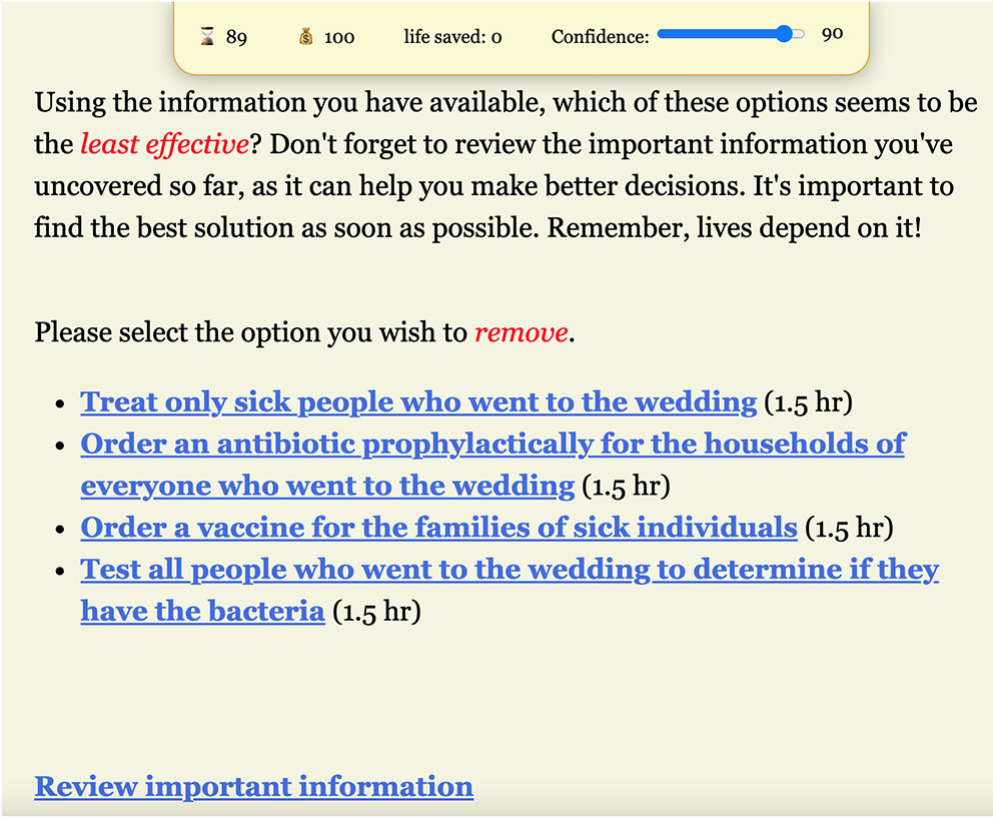
Screenshot of a decision point in the prototype. Players are presented with a short quantitative research finding and then asked to initiate a decision about intervention through a process of elimination. Focus group participants identified such processes as unnecessarily indirect and time-consuming. Our next steps will reduce the number of decision points, such that players will see all research findings at once and proceed to select the most evidence-supported decision.

The usage of an app for solving public health challenges may be relatively new in Liberia and as such features that are perceived as difficult may impact acceptability of the app. Participants emphasized the importance of simplifying the app to allow easy navigation by individuals with different levels of education and technological skills (Figure 2). Participant feedback suggested that there was interest in games that are easy-to-use. The prototype scenario required intermittent instructions from focus group facilitators to explain the game (since it was not necessarily straightforward or easy-to-use) and that interrupted the flow.

“*...I think the app should also try to limit the options it provides the users. The more options you give a person, it confuses him/her as to which one to take.” (Participant 5, Focus Group 3)*

“*I was fortunate to head a focus group discussion on community health workers, community health assistants, community health services that we had in all of the communities in rural Liberia. So, they had this app on the mobile phone that they could use……..it's made in a way that even if you do not go to school, you can work with it and treat somebody. So, for instance, if you see this person maybe an under 5 and, the app will ask you question; the person name, what are the situation the person is presenting with? Once you put, for instance if you put let's say fever, the app is going to take you on a straight path way.” (Participant 2, Focus Group 2)*

“*No, … clear options. If I choose the wrong option, let the app say this is wrong and let me think again. Because without an answer, it will be hard for me., I am suppose to save life.. it's real. We are only practicing now, but it's going to be a real life situation. We can't be doing real life situation with this thing where scatter.” (Participant 2, Focus Group 2)*

“*At the start it wasn't clear, I was totally off like what am I doing here. So, until you saw at a point she had to pause and maybe go over everything. So from the beginning it wasn't clear. So, when it later went further, It later got clear from the mini game.” (Participant 2, Focus Group 2)*

### Theme 4: Other Interactions With Games and Mobile Apps

Participant feedback demonstrated that players had not had much prior interaction with mobile games, particularly educational games or phone-based interactive applications. This was evident in references made to other apps that were not game-like but more purposeful with clear utility. For instance, comparisons were made to Kobo Collect, GPS coordinate-generating programs, and a Google search.

“*The features of the app be similar to that of the kobo collect, direct pathway, easy to use and no complication.” (Participant 1, Focus Group 1)*

“*a GPS coordinate should be added to enable the work to go on easily. The GPS is important for participants to be recorded before one participant be interviewed more than once.” (Participant 7, Focus Group 1)*

“*The search app, like the way we can go on google and get information, as fast as possible. You can compare it with any app, but I will want for it to be like search engine or google. I will like, I will recommend that the app be fast.” (Participant 3, Focus Group 3)*

### Theme 5: Engagement Factors

Participants' lack of prior engagement with mGBL also led to conflicting feedback, with some suggesting the game aspect was a distraction in the context of serious problem-solving when lives were at stake and others suggesting that the minigames were interesting and fun components of the scenario.

“*The app is great, but the aspect of giving options so that one of the actions (test guests, interview guest and test environment) to be taken is not that clear. The game should not have options whiles in the field because you want to make real time decision, not to play puzzles. The app should instruct you on what to do and what not to do.” (Participant 6, Focus Group 1)*

“*One, I think with the game, it makes learning interesting, it's fun. Yee So, you can, you can almost like want to play a normal game and you do it simply using your phone.” (Participant 1, Focus Group 2)*

Participants could relate to the time, money, and lives lost metrics and included them in their decision-making process. It is important to consider realistic, relatable, context-appropriate parameters in the design process.

“*I think the time is much more interesting because it keeps you on your toes. If I have three days to make a decision, I can go pass around and be doing stuff. But if I have a limited time, it means I have to make a decision within the time frame. (Participant 1, Focus Group 2)*

“*Yes so, for interviewing the guest there's no cost attached. So if we find the actual person, then we can just use our money and test to get that part. So it's better we save our money and get the information from them to say for the right purpose instead of just wasting the money.” (Participant 1, Focus Group 2)*

“*I learned from the scenario that whenever there is an outbreak, you have limited resources (time and money) to make decision. The best way to approach is to research and come out with the best possible decision/recommendation for action, rather than taking immediate action (without evidence) and spending more money on issue that could cost less if action taken was based on evidence.” (Participant 3, Focus Group 1)*

## Discussion

Focus group feedback about the *Figure It Out* prototype demonstrated enthusiasm for learning more effective, evidence-driven decision-making at the public health frontlines but also revealed that the current mechanisms used for learning were inconsistent with participants' expectations for the app specifically and with their interests in general. Participants expressed a preference for straightforward approaches demonstrating the correct path rather than allowing for mistakes that would send them in a less-direct path. This feedback elucidates interesting cultural differences between the game development team and end users, not unlike experiences of other transnational, cross-cultural projects (36, 37). During development, the designers did not want to make the game too simple by presenting unidirectional correct/incorrect decision paths. The goal was to encourage the player to explore different possible courses of action and learn from the consequences of incorrect action. The Western educational system reflects the concept that failure is a step on the path to knowledge and encourages creative problem-solving (38, 39). Failure should be seen as the opportunity to learn from a mistake and encourage new attempts—repeated play throughs—to find a better solution. Players from other educational backgrounds can find this process confusing and defeating, as in many non-Western cultures, grading and success in school is based on performing well on assessments.

At a higher level, the feedback affirmed that efforts promoting research utilization in public health through evidence-driven decision-making must be context-sensitive. Exploration and failure were clearly perceived by the focus groups as risky and inappropriate in scenarios in which human lives were at stake. The desire for pointed instruction also reiterates the fact that the luxury of choice often does not exist during public health practice. In resource-constrained settings, lack of adequate human, financial, and other resources—such as laboratory equipment and reagents, reliable transportation, and internet access—has direct implications for whether or not the “correct” or “ideal” path can be chosen during problem-solving. It also has the consequences of deprioritization of research, in the context of competing priorities, and thus poor awareness of the utility of research-generated evidence. Building such awareness for the utility of quantitative research is an overarching goal of the app and it warrants a stronger understanding of how and why potential users are skeptical and/or unaware. This has shaped our plan for the ongoing design process. Our next steps will firstly involve making responsive edits to the Twine prototype. In addition, we will solicit more explicit information from the UL SOPH MPH team about what skills for identifying and applying relevant research are lacking and desired as well as what gaps in understanding about quantitative research concepts (e.g., statistical significance, effect size, outcome measures) exist.

**Figure 3:**
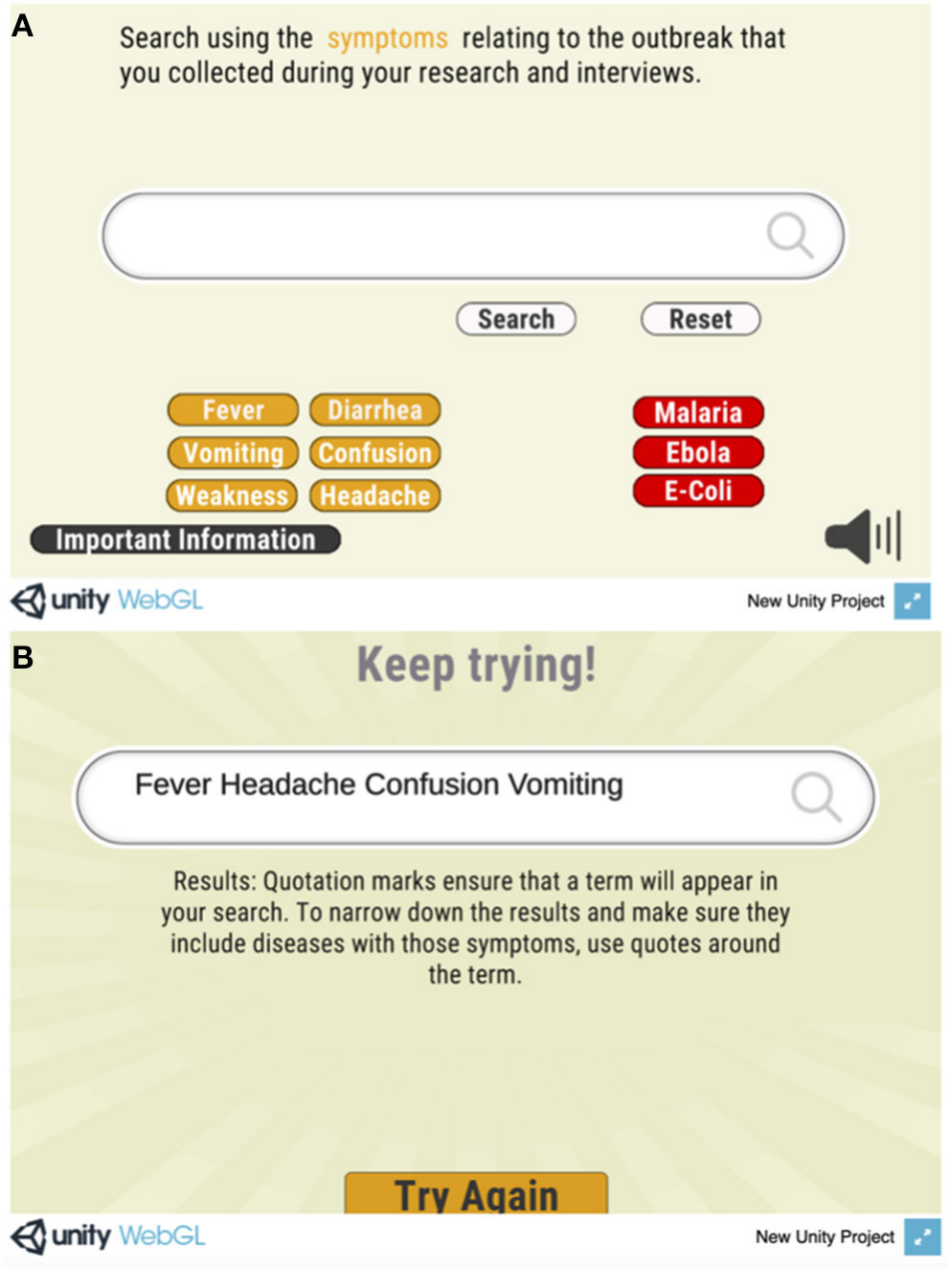
Sequence from the Search Savvy Minigame. In the original prototype, players are prompted to first search for key symptoms identified in the epidemiological investigation **(A)**. They then receive feedback to search again, this time using quotation marks (applied through the use of a button labeled “Require” in the second step) around critical search terms that must be included in the search **(B)**. To address feedback, the team has suggested that the first screen of the minigame contain the “Require” button, initially with flashing color and a pop-up hint encouraging players to click “Require” before selecting each symptom.

Edits to the prototype will be undertaken to reflect the feedback of the focus group participants and UL SOPH research team. Several changes will be intended to ensure that the game is more engaging, relatable and understandable for the users. For instance, an introductory screen will emphasize the overarching objective of the app and encourage users to consider how decisions based on evidence may often yield better results rather than decisions solely focused on time and money (Figure 1). All text, including summaries of scenarios and overviews of evidence, will be revisited to reduce wordiness and time spent reading (vs. interacting). Where possible, symbols and visual representation will be used to replace text. Moreover, the minigames will be modified to demonstrate internet searching skills and then have users go through guided practice, rather than teaching through corrective feedback (Figure 3). Similarly, both visual and verbal hints will be introduced ahead of some decision-making points in the scenario to reduce probability of users taking the less desirable path.

Ahead of outcome evaluation, a more participatory design process (40) will be undertaken in the next phase of the app development. Going forward, the design team will include the four MPH research assistants. The Liberia team will make regular contributions to the design process and lead formative next steps. For instance, the students will work with programmatic and research leadership at the UL SOPH to define learning objectives for different phases of the app (e.g., after the first three scenarios, after the first five scenarios, after the 10 total scenarios). They will identify concrete skills of interest to them as we work toward those objectives identified thus far. After any changes are implemented in the app, the student researchers will be asked about whether the changes are aligned with local learning styles and game experience (both in terms of engagement factors and technological capacity). In parallel, a larger working group will be assembled of students, faculty, and community members associated with the UL College of Health Sciences, in which UL SOPH sits, to elicit feedback at critical points in the development phase. This will also allow for testing of features and content with a more representative group, ahead of formal evaluation. Lastly, our results from the present study suggest that the target users have limited experience with entertainment or educational applications and instead use apps for work-related purposes, such as field data collection. For the latter, employers often provide devices with the application already installed. As such, a usability framework for the context will be developed for subsequent evaluation in order to reflect the entirety of the process of accessing the game on an app store, downloading on limited bandwidth network, and independent interaction with the game with built-in tutorials about both instructions for interaction, types of features and learning approaches, and accessing support from the research team. We will aim to therefore contribute a new conceptual resource to the field that accounts for limited prior experience with mGBL specifically and mobile applications in general, in part due to constraints around access to internet and ownership of individual devices. Once the prototype scenario is fully developed and locally vetted and evaluation tools have been developed, ideas for additional scenarios will be solicited from public health practitioners in Liberia and elsewhere in the West Africa region.

Ongoing capacity-building around the conduct and utilization of research is essential in promoting sustainable development efforts (41, 42) and, as such, effective novel methods, such as mGBL, to enhance such learning require exploration and adaptation for meeting local needs must be developed. The use of mGBL to accomplish educational goals in the geographic areas of interest has unique challenges and opportunities. Challenges to diffusion of mGBL, including issues around acceptability and accessibility, warrant an understanding of local technology and of the extent to which mGBL influences the participants' learning performance and motivational effects (43, 44). Despite these salient challenges, mGBL as an approach to teaching research utilization in public health practice offers opportunities to immerse users *in situations* that would have otherwise been resource-intensive, inappropriate due to sensitivity of information being collected, or high-risk in terms of health and safety (45). In summary, our findings have offered the following specific insights into the use of mGBL for informing evidence-based, public health practice in resource-constrained settings:

1. It is important to consider how local educational expectations will interact with the concept of using playfulness and exploration to learn. It could easily be counter-intuitive and thus the game needs to be introduced or explained better at the start to prepare players.
2. Games that involve life or death decision-making should reflect that seriousness in the game design and not make players feel as though they are playing with people's lives.
3. Provide focused, context-specific guidelines so that players feel like they learned useful, actionable information to inform their actions outside the game.
4. Use the game experience (and technical abilities of digital media) to collect information from players about skills they lack and would like to learn.

The *Figure It Out* app is envisioned to promote more awareness and exploration of quantitative research evidence in public health practice across resource-constrained settings, such as West Africa. We aim to appropriately use the app to teach why decisions backed by research evidence could ultimately lead to more cost-effective, time-efficient, and life-saving decisions across different relatable scenarios, even if that evidence comes from other settings. Going beyond the specifics of any one situation or task, the app will offer virtual opportunities to practice the logic and critical thinking skills needed for effective research utilization in realistic scenarios. Incorporating feedback gained from participatory design processes will increasingly ensure that the app addresses local interests and needs to optimize utility, usability, and acceptability.

## Data Availability Statement

The original contributions presented in the study are included in the article/Supplementary Material, further inquiries can be directed to the corresponding author/s.

## Ethics Statement

The studies involving human participants were reviewed and approved by University of Liberia PIRE IRB. The participants provided their written informed consent to participate in this study.

## Author Contributions

LS, EB, MI, DK, PS, and QL: conceptualization. JS, HK, AC, and JZ: focus group testing. JS, HK, AC, W-MH-G, and LS: thematic analysis. DK, EB, QL, PS, LD, EH, and MM: software development. DK, EB, LD, EH, and MM: software testing. EB and LS: supervision. LS, JS, HK, and AC: writing original draft. EB and MI: editing of manuscript. All authors contributed to the article and approved the submitted version.

## Conflict of Interest

The authors declare that the research was conducted in the absence of any commercial or financial relationships that could be construed as a potential conflict of interest.

## Publisher's Note

All claims expressed in this article are solely those of the authors and do not necessarily represent those of their affiliated organizations, or those of the publisher, the editors and the reviewers. Any product that may be evaluated in this article, or claim that may be made by its manufacturer, is not guaranteed or endorsed by the publisher.

## References

[1] Lin WC Ho JY Lai CH Jong BS. Mobile game-based learning to inspire students learning motivation. In: 2014 International Conference on Information Science, Electronics and Electrical Engineering Sapporo, Japan (2014).

[2] Tan WH. Design, Motivation, and Frameworks in Game-Based Learning. Hershey, Pennsylvania: IGI Global (2018).

[3] Marcus-Quinn A Hourigan T. Handbook for Online Learning Contexts: Digital, Mobile and Open: Policy and Practice. Cham, Switzerland: Springer Nature (2021).

[4] Costabile MF De Angeli A Lanzilotti R Ardito C Buono P Pederson T. Explore! possibilities and challenges of mobile learning. In: Proceeding of the Twenty-Sixth Annual CHI Conference on Human Factors in Computing Systems - CHI ' Florence 08 (2008).34463629

[5] Porter G Hampshire K Milner J Munthali A Robson E de Lannoy A. Mobile phones and education in sub-Saharan Africa: from youth practice to public policy. J Int Dev. (2016) 28:22–39. 10.1002/jid.311625855820

[6] Shonola SA Joy MS Oyelere SS Suhonen J. The impact of mobile devices for learning in higher education Institutions: Nigerian Universities Case Study. Int J Mod Educ Comp Sci. (2016) 8:43–50. 10.5815/ijmecs.2016.08.06

[7] Rosenberg S. Basic Mobile Phones More Common Than Smartphones in Sub-Saharan Africa. Available online at: https://www.pewresearch.org/global/2018/10/09/majorities-in-sub-saharan-africa-own-mobile-phones-but-smartphone-adoption-is-modest/ (accessed August 24, 2021).

[8] Ferreira SM Gouin-Vallerand C Hotte R. Game based learning: a case study on designing an educational game for children in developing countries. In: 2016 8th International Conference on Games and Virtual Worlds for Serious Applications (VS-GAMES) Barcelona, Spain (2016).

[9] Bertozzi E Bertozzi-Villa A Kulkarni P Sridhar A. Collecting family planning intentions and providing reproductive health information using a tablet-based video game in India. Gates Open Res. (2018) 2:20. 10.12688/gatesopenres.12818.1 29984358 PMC6030399

[10] Yadav AK Oyelere SS. Contextualized mobile game-based learning application for computing education. Educ Inf Technol. (2020) 26:2539–62. 10.1007/s10639-020-10373-3

[11] Omoregbe NA Azeta AA Adewumi A Omotoso OO. Design and implementation of yoruba language mobile tutor. In: Proceedings of EDULEARN14 Conference. Barcelona, Spain (2014). p. 3942–47.

[12] Oyesiku D Adewumi A Misra S Ahuja R Damasevicius R Maskeliunas R. An Educational Math Game for High School Students in Sub-Saharan Africa. In: Florez H, Diaz C, Chavarriaga J, editors. Applied Informatics ICAI 2018. Communicaions in Computer and Informaion Science. Cham: Springer (2018). p. 228–238. 10.1007/978-3-030-01535-0_17

[13] Behague D Tawiah C Rosato M Some T Morrison J. Evidence-based policy-making: the implications of globally-applicable research for context-specific problem-solving in developing countries. Soc Sci Med. (2009) 69:1539–46. 10.1016/j.socscimed.2009.08.006 19781839

[14] World Health Organization. Strengthening Public Health Laboratories in the WHO African Region: A Critical Need for Disease Control. Available online at: https://www.afro.who.int/publications/strengthening-public-health-laboratories-who-african-region-critical-need-disease (accessed August 28, 2021).

[15] Woolhouse MEJ Rambaut A Kellam P. Lessons from Ebola: improving infectious disease surveillance to inform outbreak management. Sci Transl Med. (2015) 7:307rv5. 10.1126/scitranslmed.aab0191 26424572 PMC5819730

[16] Shamasunder S Holmes SM Goronga T Carrasco H Katz E Frankfurter R. COVID-19 reveals weak health systems by design: why we must re-make global health in this historic moment. Glob Public Health. (2020) 15:1083–9. 10.1080/17441692.2020.1760915 32352911

[17] Mwendera CA de Jager C Longwe H Phiri K Hongoro C Mutero CM. Facilitating factors and barriers to malaria research utilization for policy development in Malawi. Malar J. (2016) 15:512. 10.1186/s12936-016-1547-4 27760552 PMC5070004

[18] Woolfrey L. Knowledge Utilization for Governance in Africa: evidence-based decision-making and the role of survey data archives in the region. Inf Dev. (2009) 25:22–32. 10.1177/0266666908101261

[19] Jao I Kombe F Mwalukore S Bull S Parker M Kamuya D. Involving research stakeholders in developing policy on sharing public health research data in Kenya: views on fair process for informed consent, access oversight, community engagement. J Empir Res Hum Res Ethics. (2015) 10:264–77. 10.1177/1556264615592385 26297748 PMC4548475

[20] Inguane C Sawadogo-Lewis T Chaquisse E Roberton T Ngale K Fernandes Q. Challenges and facilitators to evidence-based decision-making for maternal and child health in Mozambique: district, municipal and national case studies. BMC Health Serv Res. (2020) 20:598. 10.1186/s12913-020-05408-x 32605564 PMC7329398

[21] Marjanovic S Hanlin R Diepeveen S Chataway J. Research capacity-building in Africa: networks, institutions and local ownership. J Int Dev. (2013) 25:936–46. 10.1002/jid.287034216733

[22] Chu KM Jayaraman S Kyamanywa P Ntakiyiruta G. Building research capacity in Africa: equity and global health collaborations. PLoS Med. (2014) 11:e1001612. 10.1371/journal.pmed.1001612 24618823 PMC3949667

[23] Bozio CH Vuong J Dokubo EK Fallah MP McNamara LA Potts CC. Outbreak of Neisseria meningitidis serogroup C outside the meningitis belt-Liberia, 2017: an epidemiological and laboratory investigation. Lancet Infect Dis. (2018) 18:1360–7. 10.1016/S1473-3099(18)30476-6 30337259 PMC6545567

[24] Twine an Open-Source Tool for Telling Interactive Nonlinear Stories. Available online at: http://twinery.org (accessed September 24, 2021).

[25] Unity Technologies Unity. Available online at: https://unity.com/ (accessed September 24, 2021).

[26] Dilshad RM Latif MI. Focus group interview as a tool for qualitative research: an analysis. Pak J Soc Sci. (2013) 33:191–8. http://pjss.bzu.edu.pk/website/journal/aricle/5e9756b9cf255/file/5e9757486e8e3/view

[27] Carlsen B Glenton C. What about N? A methodological study of sample-size reporting in focus group studies. BMC Med Res Methodol. (2011) 11:1–10. 10.1186/1471-2288-11-26 21396104 PMC3061958

[28] Davis AM James RL Curtis MR Felts SM Daley CM. Pediatric obesity attitudes, services, and information among rural parents: a qualitative study. Obesity. (2008) 16:2133–40. 10.1038/oby.2008.312 18551114 PMC2701507

[29] Francis SA Katz ML. The HPV vaccine: a comparison of focus groups conducted in South Africa and Ohio Appalachia. Matern Child Health J. (2013) 17:1222–9. 10.1007/s10995-012-1116-6 22930347 PMC3799762

[30] Cowley B Heikura T Ravaja N. Learning loops–interactions between guided reflection and experience-based learning in a serious game activity. J Comp Assist Learn. (2013) 29:348–70. 10.1111/jcal.12013

[31] Gris G Bengtson C. Assessment measures in game-based learning research: a systematic review. Int J Serious Games. (2021) 8:3–26. 10.17083/ijsg.v8i1.383

[32] Mitgutsch K Alvarado N. Purposeful by design? A serious game design assessment framework. In: Proceedings of the International Conference on the Foundations of Digital Games (FDG '12). ACM, New York, NY, USA (2012). p. 121–8.

[33] Rashid T Asghar HM. Technology use, self-directed learning, student engagement and academic performance: examining the interrelations. Comp Hum Behav. (2016) 63:604–12. 10.1016/j.chb.2016.05.084

[34] Nowell LS Norris JM White DE Moules NJ. Thematic analysis: striving to meet the trustworthiness criteria. Int J Qual Methods. (2017) 16:1609406917733847. 10.1177/1609406917733847

[35] Vaismoradi M Turunen H Bondas T. Content analysis and thematic analysis: implications for conducting a qualitative descriptive study. Nurs Health Sci. (2013) 15:398–405. 10.1111/nhs.12048 23480423

[36] Chen A-Y Mashhadi A Ang D Harkrider N. Cultural issues in the design of technology-enhanced learning systems. Br J Educ Technol. (1999) 30:217–30. 10.1111/1467-8535.00111

[37] Arani MRS Shibata Y Sakamoto M Iksan Z Amirullah AH Lander B. How teachers respond to students' mistakes in lessons: a cross-cultural analysis of a mathematics lesson. Int J Lesson Learn Stud. (2017). 6:249–67. 10.1108/IJLLS-12-2016-0058

[38] Heimbeck D Frese M Sonnentag S Keith N. Integrating errors into the training process: the function of error management instructions and the role of goal orientation. Pers Psychol. (2003) 56:333–61. 10.1111/j.1744-6570.2003.tb00153.x

[39] Dyre L Tabor A Ringsted C Tolsgaard MG. Imperfect practice makes perfect: error management training improves transfer of learning. Med Educ. (2017) 51:196–206. 10.1111/medu.13208 27943372

[40] Zachary WW Michlig G Kaplan A Nguyen N.-T. Quinn CC. Participatory design of a social networking app to support Type II diabetes self-management in low-income minority communities. Proc Int Symp Hum Factors Ergon Healthc. (2017) 6:37–43. 10.1177/2327857917061010 31157286 PMC6542278

[41] Chataway J Smith J Wield D. Science and technology partnerships and poverty alleviation in Africa. Int J Technol Manag Sustain Dev. (2005) 5:103–23. 10.1386/ijtm.5.2.103_126161545

[42] Nuyens Y. No Development Without Research: A Challenge for Capacity Strengthening. Global Forum for Health Research Geneva, Switzerland (2005).

[43] Egenfeldt-Nielsen S. Overview of research on the educational use of video games. Nord J Dig Literacy. (2006). 1:184–213.

[44] Huang YL Chang DF Wu B. Mobile game-based learning with a mobile app: motivational effects and learning performance. J Adv Comp. (2017). 21:963–70. 10.20965/jaciii.2017.p0963

[45] Prensky M. Teaching Digital Natives: Partnering for Real Learning. Thousand Oaks, California: Corwin Press (2010).

