# Supplementary material for: Participatory Design and Process Testing to Optimize Utility, Usability, and Acceptability of a Mobile Game for Promoting Evidence-Driven Public Health Decision-Making in Resource-Constrained Settings
Source: Front Digit Health. 2022 Jan 4;3:788557. doi: 10.3389/fdgth.2021.788557 (PMC8763845; doi:10.3389/fdgth.2021.788557)
Supplement: Supplementary file 1 [file Data_Sheet_1.docx]

Supplementary Material

# Supplementary Data - Transcript from Focus Group 1

**After Minigame 1**

Participant five (5)- The new thing learned from the app was the minigame (Search Savy). My problem was to include symptoms and exclude the disease condition. I think every symptoms associated should have a certain condition attached, not to exclude or include diseases for the scenario under investigation.

Participant five (5)- With the minigame which is new, field work is to move out those diseases that are not associated with a symptoms, the app should consider such feature.

Participant six (6)- Looking at the drill, it was not clear as to when to put in the require before punching in the symptoms or minus sing a disease. The app should be very clear.

Participant four (4)- When a person has a limited resource, he/she is able to put their money and time into proper use with the minigame.

Participant seven (7) – The try again option in the game should not be allocated to this app.

Participant six (6)- The entire drill especially the minigames where one has to type in the symptoms collected from the investigation, it was not clearer as when to click **require**. So going back and forth makes the game boring and less interesting to the participant.

Participant six (6)- I recommend that in order to make the game more interacting and interesting with respect to the minigame, there should be a clearer instruction to click **require** first before typing in any symptom, so as to avoid going back and forth.

Participant six (6)- The entire drill especially the minigames where one has to type in the symptoms collected from the investigation, is not clear as when to click **require**. So going back and forth makes the game boring and less interesting to the participant.

**After Minigame 2**

Participant two (2) Health practioners are problem solvers, and want to figure out what cause a particular health issue. With that regard, the app makes one to understand that it is advisable to do extensive research when an issue arises so as to be able to acquire more knowledge/idea on how to eradicate or curtail the problem by making informed decision based on the knowledge acquired (research evidence).

Participant (5) – Front liners are using the app to gather information in the field, the outbreak in the field might be different from what will be seeing in the lab. The app should be open for other details information.

Participant (7) – If we decide to Interview guest, a GPS coordinate should be added to enable the work goes on easily. The GPS is important for participants to be recorded before one participant be interviewed more than once.

Participant one (1)- As surveillance officer in the field trying to solve an outbreak, there is limited time to type in the symptoms in the search engine, so there is a need that the app be clear on the kind of symptoms to be searched that will yield the result you hope to obtain. I will like to play the minigames again because it was helpful the first time.

Participant one (1)- Let me re-emphasize what participant six (6) said about the **required** option within the minigame. So, I will want to recommend that there be a clearer instruction that says “before you type in any symptom, you must first click require” to avoid the back and forth or the try again option which makes the game boring.

Participant two (2)- There’s not a need to have required button in this second game to put in symptoms and exclude a condition, rather to tell you try again. One should be able to move their way through haven played the first game and went through this same procedure.

**General Feedback**

Participant six (6)- The scenario was quit helpful but timing should be allocated to every aspect of it. This will allow you to know how fast you should be.

Participant three (3)- I learned from the scenario that whenever there is an outbreak, you have limited resources (time and money) to make decision  . The best way to approach is to research and come out with the best possible decision/recommendation for action, rather than taking immediate action( without evidence) and spending more money on issue that could cost less if action taken was based on evidence.

Participant three (3) – One has limited time to enter those symptoms that are associated with a condition, there should be a clue in the app that will lead you to the next option/ decision to take.

Participant four (4) - The app is an interesting and helpful tool in solving Public health issues. For instance, when a public health expert is in the field investigating an issue with limited resources (time and money), the app helps you think fast and wisely to come out with informed decision based on acquired information/idea from the research carried out.

Participant two (2) -The app is good and I learned a lot from the app. One of those things learnt is that when an investigation is ongoing in the field, the best thing is to research before making decision. With regard to the minigame, I recommend that once you type in the symptoms in the search bar, the only result that should be seen is the disease related to those symptoms.

Participant six (6) -The app is great, but the aspect of giving options so that one of the actions (test guests, interview guest and test environment) to be taken is not that clear.

The game should not have options whiles in the field because you want to make real time decision, not to play puzzles. The app should instruct you on what to do and what not to do.

Participant five (5)- The most interesting part of the game is the decision making process (the four public health recommendations). It is clearly stated that choosing any of the recommendations could either lead to saving lives or losing lives.

Participant one (1)- Making real time decision and thinking critically was the most interesting part.

Participant three (6) – The app should have some symbols that will tell at the end of the research as to whether they killed, or save lives.

The least interesting thing is time consuming. The information in the app should be summarized and not to be explained in literature.

Participant one (1)- The idea of going through so many steps before making decision did not seem interesting.

Participant six (6)-The scenario was helpful, and it makes you think out of the box. I’ll recommend that time should be allocated to every decision that one will make through out the game so as to make you think faster.

Participant one (1)- The features of the app be similar to that of the kobo collect, direct pathway, easy to use and no complication. I’ll also recommend that the **view important information** at the bottom should not be seen at the bottom of every page especially the ones it is not use for.

Participant six (6)- Some added features with respect to the options(Test Guests, Interview Guests, Test Environment), specific symbols should be seen as a means to illustrate.

Eg, Test Guest should have a microscope; Interview Guest should have maybe a microphone.

Participant two (2)- I’ve understood that when an investigation is ongoing in the field, there’s a need to go deeper in your search before making decision. When those symptoms are punched in, the disease should pop up.

Participants one (1)– I’m actually confused over; Reading Terms & Important information.

Followed by Participants 3, 6, & 7with the same confusion.

Participant (2)- Households should be added to the app so they can’t be reached more than once.

Participant (6) – The option of getting name of participants should not be included in this app.

Participant (4)- The Critical thinking aspect was interesting.

Participant (6)- If one of the options to make a decision is removed, that same option shouldn’t be seen on the list of the next options.

Participant (4)- A place for second chance should be given in the decision making process.

Participant (1)- I will like for this app to resemble kobo collect. They both will collect real time data.

Participant (3)- At the end of the process, there should be a GPS coordinate to tell who wants, where, and what next to do.

Participant (1)- Reviewing important information shouldn’t be seen at the bottom of every page, especially those pages that they are not used.

Participant seven (7)- I have no interest in the length of time it takes to come up with a real decision. I think more lives will be lost before a decision is reached.

# Supplementary Data - Transcript from Focus Group 2

After the introduction by the facilitator and reading of the scenario, will you act, or want to read more information?

**Participant#1**. I just want to know about investigation to find out at the weeding looking at the environment the 25 persons were in or whether they ate together base on getting information of what the 25 persons had in common.

Participant#4. Yea number 4, just what he said, we will try to do some follow up and investigate how many persons attended that particular wedding, what they ate during the wedding.

**Participant#4**.  we can name this group discussion focus group 2.

Participant#3. Yea, number 3. Number 3. I think you‘ll view information to find at first the cause of those diseases or the cause of them coming down with this headache or this fever and symptoms because you cannot just go forward without bringing or without getting feedback for cause or what cause can do that. So, before doing anything, you know they were suffering from other we don’t know.

**Participant#2**. Ok, number 2 so, because you had read important information as to what happened, you still need to do some test to confirm what causing the death, and what with that test I think you also need to start maybe putting in some health measures to prevent other people around from falling sick as well.

**Participant#1**. Yes, yea, I will take reviewing important information because of it.

**Participant#2**. So, number 2.  I think we want to review important information.

**Participant#1**. So, for, for me, I will think will more research interns of the 25 persons when they left from the wedding they went home in that, maybe each person going to their home. Was they exposed? Other people in their home, was they exposed? Maybe they were exposed to narrow the factors whether it’s a, whether it’s a virus that can spread through air or can be spread through food or is it through water. So, we can move that other part from there.

**Participant#2**. So, excuse me before we go further, so when you give the scenario, we’re suppose to pick from the option that will come?

**Participant#3.** Number 3. The one that is displaying on the board right?

**Participant#3**. oo ok, I understand.

**Participant#1**. Ooh okay, alright. So we’re going to take decision base on reading?

**Participant#4**. Act. Because it has, it has already been caused right? So, if you say you doing research or still like searching, who knows what may happen? Even the 25 persons you know already, who knows if it will extend to more than 25. So aye, for me, you act to at least make it to not go to others.

**Participant#1.** One,

**Participant#2**. So,

**Participant#1**. One, I think we will search because the symptoms that the person is showing, is a broad symptoms that in, in, incorporation lot of different diseases. It could be a parasitic disease; it could be like the fungus, or the ones we listed before. So, we have to search or to narrow it down.

**The first Minigame**

**Participant#2**. symptoms for the mini game?

**Participant#2**. headache, weakness, mental confusion and vomiting.

**Participant#4**. can you please go over it again, the previous, the, the game!

**Participant#4.** I understand that, it’s ok.

**Participant#2**. Ok, number 2 so, I think I’ve seen or I’ve heard about some other apps that surveillance officers used or the CHA’s and CHSS’s but for this one, it helps diagnose. So for instance, if you are having this outbreak, it helps , maybe tell you what causing a particular outbreak, what causing XYZ signs and symptoms in so, so and so. This app I think the diagnostic part is awesome for me.

**Participant#3.** to add up to what she said with this app, it make easier for you to figure out like you have Ebola you have all of these other malarias. But with this app, it help you like, if you already see all of the symptoms, it help you to go directly to the disease that is showing the symptoms, it help you to bring it up quicker. So, with this app, I think it will, it will be awesome, yeah.

**Participant#5**. with this app, it will make the work easier, it make it faster because with this they already given you a diagnoses of the illness with the weakness. It will able make, the, the app make the work easier and faster.

**Participant#2**. Another thing # 2, the options serves as direction. So, for instance, if you are supposed to go this riot, once you click the wrong button it will take you back and you’ll go the right way.

**Participant#3.** so, it’s almost like yes/ no if you in one direction.

**Participant#1**. Yea, One. I think the instructions there are very clear. It helps to find out the step by step methods that requires the results.

**Participant#2.** At the start it wasn’t clear, I was totally off like what am I doing here. So, until you saw at a point she had to pause and maybe go over everything. So from the beginning it wasn’t clear. So, when it later went further, It later got clear from the mini game.

**Participant#1**. I can I write down my recommendation? Yes I, I think the instructions are clear, it helps you to follow the step method to get what is required.

**Would you like to play the game?**

**Participant#2. Number 2**. Yes

**Participant#3**. Number 3. I will say yes.

**Participant#5**. Number 5. I will, I will say yes.

**What’s about the game you will like to play often?**

**Participant#1**. One, I think with the game, it makes learning interesting, it’s fun. Yee So, you can, you can  almost like want to play a normal game and you do it simply using your phone and you do it like;……….even when you feeling sick, you can play the game and be able to get a certain sign and your personal status at the time.

**What can be done to make the game better or more interesting as seeing it earlier?**

**Participant#1**. One.  yes , I think , there are others….. For example like the options are squeezed  into writing. I think if we can draw it from writing and put it in specific column that people can be able to click on it directly instead of just fighting, fighting your way like for example where the game is waiting you have to go all the way through try again and remove other things, but if the column is there, you can just click and start.

**Confident Bar**

**Participant#1**. yea we are 100% confident

**Participant#1**. yea why not , I think we can do that and save the lives that we want as playing the game because timing will help us to bring out to make rational decision because saving lives require time. Every lil decision you make or delay in making decision could kill someone or save their life.

So, with the timing aspect there, the money aspect is like creating an interest in making it more interesting where you can be able to earn points and stuff like that…….. I think…… you know? Just like that.

**What’s on the confidence bar that is much more interesting?**

**Participant#1**. Aye one, I think the time is much more interesting because it keeps you on your toes. If I have three days to make a decision, I can go pass around and be doing stuff. But if I have a limited time, it means I have to or make decision within the time frame.

**Participant#2**. So, number 2, I will take saving life because it, it also has a connection with time because if you have to save a person life it has the stuff with time. For instance, maybe 30 minutes to work to do something, to do an intervention for this person life to be saved, so………. All talk about time.

**Participant#3**. I say  saving life to time.

**After Minigame 1**

**Participant#1**. One, one.  I will like to interview the guest to find out, to get the actual information from them because we will know more about that sickness, where they went. Whether they ate any other food, any other place within that time from while the wedding was going on or when they arrived, they remained there ate what they ate there, so we can have all the information.

**Participant#3**. Yes, number 4, 3. Hahaha, number 3. I will say test the environment, yes. The reason is that it was within that environment where they encounter all of these problems. It was there where they had the food, where they had the drinks. What so ever it was within that environment so, I will have, I will test the environment. Yes, I seeing the cost.

**Participant#2.** So well, number2, I will test the quest. Even thou it is quite a lot of money, the …….. it will take a step further because I ‘m already ask a lot of people and I already  like narrow it down so, I will test to know what causing the particular situation that is happening.

**Participant#4**. Number four. Act, at this point on the prior knowledge that I have. It has already been caused, if you say you are doing research or searching, who knows if it will extend from more than 25 persons to more. So for me, I think I will act.

**Participant#4**. so can I ask the ………for this, I will say interview the guest because with that we get prompt intervention. yea, you have limited hours, so, I prefer you interview the guest.

**Participant#1**. yes so, for interviewing the guest there’s no cost attached. So if we find the actual person, then we can just use our money and test to get that part. So it’s better we save our money and get the information from them to say for the right purpose instead of just wasting the money.

**Participant#1**. Interview the guest.

**Participant#4**. Interview the guest….. Yes.

**Participant#2**. Interview the guest.

**After interviewing the guest**

**Participant#2**. so, number 2, I think we want to do view important information.

**Participant#1.** For me, I will take the results oo, because moving forward….

**Participant#1**. One, For me, I will take result oo. Yea, I will take the results because we are already focusing on the 25 person because we…… those are the contagious people now, we need to pay more attention to… So, we need to get the results from them. Yes, that’s what I ‘m saying. Get the information from them, the 25 persons because it’s from that information then we will know we will know whether they ate together, what specific food they ate as it relates to the information provided. Then we can know whether if its air born, water born.

**Participant#3**. so, the first thing they have already interview guest right, so what we are responsible for is to get the results.

**After the result (should we play another game?).**

**Participant#2**. Number 2, I think so

**Participant#1**. One, for me, our clock is taking, I will like for us to test the quest.

**Participant#3**. Let’s try the game.

**During/After the second mini game**

**Participant#2**. Ok, Generalize weakness, vomiting, headache.

**Participant#2**. Generalize confusion there.

**Participant#1.** Yes, generalize confusion there, they took, they took one symptoms from there in the first game.

**Participant#2**. Yea,

**Participant#1**. Symptoms could be generalized. So it was headache, vomiting, generalize weakness.

**Participant#1**. Yea, there was no confusion.

**Participant#3**. Confusion was not there.

**Most interesting part of the prototype**

**Participant#1**. One, I was, initially I wanted us to test the guest but when we played the game, I saw the options coming up on the screen as we were limiting picking the options and how restricting it to food born, water born, air born. That was interesting, when I saw that part, I was like regretting why I didn’t play the game. I was going to test guest because there was not a clear picture if I was to test or if I was going to reach to it. So, the game was interesting to me, it gave me a clear picture of what I was hoping to see.

**The most Boring part/ least important part**

**Participant#4**. Four, everything was very ok, I was not bored with anything.

**Participant#2**. For….

**Participant#1**. Yeah, one. We eliminated Ebola at the first game stage, but Ebola re-surface at the second game. I don’t really think…. Ehn, Ebola was at the first game stage and we eliminated it but it re-appear in the second stage. So, we eliminating, its returning. So…….

**Participant#2**. Yea, that’s what I wanted to say. And another thing, I think we did not. Number 2, I think because we are not the ones playing it, it was kind of like just watching it. Probably you aren’t know what you are playing to. We are just seeing.

**Participant#2.** Yeah, to play it myself. …….Yeah, yeah, yeah yea.

**In what section you got stuck/confused?**

**Participant#5.** Number 5, The place where she was selecting the symptoms. Generalize weakness,…..yeah. the symptoms, I got a bit confuse there.

**Participant#1**. Yeah one. I think interns of placing the symptoms in, in the bar, I could see you going backward and forward. So, I think the direction was not clear. Yeah, whether if you click, I think interns of, maybe I will make it my recommendation. I thick if you click, then the app can be built in the way that… like for example, I think if you click a symptoms the app suppose to press require right? Alright, I think the app, the app can be built way that….. Recommendation, once you click the symptoms, the app should compel you to tap, to tap the require before you will be able to play another one. Because if you click it twice, then you will be just in that area your time will be going. So, I think the app can be built that if you click the symptom, you are compel to press the require before you can press to another symptom.

**Participant#2**. Uhum. So with the option part, I think the option part where they have to be test guest, or test the environment. What I want to say is that this app is going to used by individuals like when you go in the field. You will not be there, she will not be there, I will choose what works for me. so, there will be issues. So everybody will have sayings pertaining to what is right, what’s the right thing to do. So everybody will be thinking of what they thought was the best. If he gives his answer, I give my and justify. So, in that field where I am. I’m going to give you mine, what I think, I’m not going to consider whether people think is right or wrong. Yep, it should be to the point. No need of compelling to do the right thing instead of at least to choose. So if, you, if you click something that is wrong, let it say this is wrong and let they give me the right thing.

There should be options or there, it…… For instance, if you were diagnosing for malaria, let it have a clear path, yeah, but if you giving me options to choose from, I’m alone right? And I got time, I get money. I will choose what works for me, I will choose what I think. I will not be considering other people’s feeling. So if, if you giving me options then you say I can’t go back.

**Participant#3**. If I got you, so you are trying to say that there will be no options, you will just go straight?

**Participant#2.** There should be options but it should take you straight to the point.

**Participant#3.** But yea, she’s working alone na,

**Facilitator#1**. No , she’s trying to say that, if you are investigating malaria, here should be path way

**Participant#2.** No, …… clear options. If I choose the wrong option, let the app says this is wrong and let me think again. Because without answer, I suppose to save life, it’s a real. We are only practicing now, but it’s going to be a real life situation. We can’t be doing real life situation with this thing where scatter.

Yeah, that’s what I’m telling your.

**Participant#1**. One. I think there’s a set standard procedure in case of investigating an outbreak. I think the app should follow that path, that pathway. So, if we are to play the game to get more information, the app should not confuse us to do something that is not really necessary. Let us just to the right procedure because from what I observed. I wanted to test the guest. But when I saw us playing the game, if I had seen that information I would have gone straight to the right thing. So, if are playing the game, lets just play the game instead of saying, creating the one where we will have to be confuse. Cos you have the re-start the app and start the whole procedure over.

**Participant#2**. So, one thing could, number 2. One thing could be maybe after a situation, we play the game to get more idea. Do another procedure, you play the game to get more idea, just like that.

**How long the game should be played for before making a decision?**

**Participant#1**. One. I want to ask a question first before I make this comment. That 65 hours they have there, is it a real time 65 hours?  Yea, I’m saying, according to the game, is it a real time 100 hours? Is it a real time, the 100 hours?

I think the time is too long to make a decision in some real 100 hours time. So, I’m thinking that, depending on…… the game should narrow It down to maybe a 15 or at most an hour. In real time at most. To make that…..Because I think the game like for example, if I were to go into a village where there is no health facility and I’m seeing the symptoms and I want to, I have a lot of drugs in my bag and I want to use the app to make a decision and I need to make that decision within the soonest possible time so that I will give the drug that will put the person maybe stablelize the person before the ambulance or what so ever can come for us to take them or take the person away. Because that means if you had health facility you could easily walk the person there for test, but then if you’re in one village maybe that 1 hour walk or 2 hours walk, you have to make a decision at that time.

**Compare this app with other research tools/ apps for improvement**

**Participant#2**. Number 2. So, I was fortunate to head a focus group discussion on community health workers, community health assistance, community health services that we had in all of the communities in rural Liberia. So, they had this app on the mobile phone that they could use. So, for the community health assistance, they are not educated, they are I think high school graduate, or in 6^th^ grade. Once you can pronounce, you can use it.

And then the app that we have, it’s made in a way that even if you do not go to school, you can work with it and treat somebody. So, for instance, if you see this person maybe an under 5 and, the app will ask you question; the person name, what are the situation the person is presenting with? Once you put, for instance if you put let’s say fever, the app is going to take you on a straight path way. Like everything connecting you to reach to where or to a point where you have to treat. And if you had like about four conditions that you’re treating in the community. So, with that, it’s easy once you don’t know as a person. You do it to ask the person and it’s done within a shorter period of time. Like you can take 5 minutes to do it and treat treat right on the spot. So, if I am. It’s called the Liberia ODK. The national community health…… They are using it. So, it is emm, because of that app, they are so you don’t get to the clinics in rural areas you see a lot of under 5  children there, so because somebody in the community there to treat the under 5 and they have their drug and everything on them. And it’s fast. Like, you can’t give it wrong answer. If you put a wrong answer there, it will take you back to re-consider what you just put in. so the, unlike this one that have maybe pathway. You have to figure out maybe it time to treat, and you might say, you will brainstorm and be long. So, let it just be straight to the point. Yea, clear cut, straight to the point. Say what you suppose to do and do it on time.

Your reduce that plenty time.

Yes, yes, I think Dr. Gray should be familiar with the app that the national community health assistance program is using.

**Participant#1**. Like in here, I think the app is just giving a clear picture of the disease. It’s not giving us any treatment format. Because, like what she was talking about, putting the child there. Because if the app is to add that part, the app is sensitive enough to look at the BMI of the person they putting the information on. Because you cannot give a 5 years old like 500 mg. that will be too much for his body weight. So, that part can be taken into consideration because of no nearby clinic. Even if you decide to add the treatment they will get back to you to know whether it is necessary to know what doses to give.

**Participant#4**. and also to what she was saying is also very important because if you know the symptoms, that the person is giving and will just do it to able to guide you through, it will be very, very important.

**Participant#1**. Yea, Before 10, 15 years ago there was no laboratory in the clinic. So maybe when you want to test for malaria test it was just this common, common treatment them. So if you go to the hospital and you give like 3, 4, 5, 6 symptoms of malaria, they give you malaria treatment.

**Participant#4.** For me, that app Anthoinet was talking about, I was in…. 4, 4, 4. I was in Rivercess before, and that particular was used on that side, I get a cousin working there with the app. Once they bring the child to her, once she get, in fact the people will look on the app and put all your information there, once you get the information, go on your app; your prescriptions, your diagnosis everything there. So, I think that app will be ok

**Participant#5**. Number 5. The features of that app…..

**Would you like to share your experience with others about the game?**

**Participant#1**. Off course, why not off course.

**Participant#2**. Number 2, number 2. This whole thing just had make more sense if we have it on our phones like we working with it. Because honestly I’m just here and looking.

But your suppose to try it on our phones, when your finish, your delete it.

**Participant#4.** For me I borrowed this person computer. I thought your were going to put it there for me to play it.

**After Minigame 2**

**Participant#3**. The steps very long, it boring to admit.

**Participant#4**. I will pick the one that say order vaccine for the family.

**Participant#4**. The least effective option to eliminate is saving live…….

**Participant#3**. Remove the one that says, test all people who went to the wedding to determine if they have the disease.

**Participant#5.** I will take the one that say, Test all people who went to the wedding.

**Participant#1**. Yea eliminate it, I will go for that one, test all people. because we get timing aspect, forget the 25 minutes. We’ll bring the whole 200 back to test them.

**Participant#1**. Yes, one. Once the other 175 persons is not showing symptoms, to test them will make us spend plenty.

**Participant#2**. So ok, at least it’ll lead to the fact that other people who are sick

**Participant#4**. Looking at everything in the scenario right? Do we have to eliminate to make progress? Then, option 5 should be removed.

**Participant#1**. Remove option 5, it won’t be necessary now.

**Participant#4.** We killed 8 persons, hahahahha.

**Participant#2.** Order vaccines for family…… yea, yea,

**Participant#3.** Order vaccines

**Participant#2.** Order vaccines

**Participant#1.** No, I thinking like for example, we were trying to for vaccine for the 25 persons, so bringing the families of that 25 persons incase that 25 persons get over 200 family members?

We coming kill people again, hahaha… so,…. But what make, as for me, I remove number one. Treat only sick persons……

See number 2, order antibiotics for households, family member and all, children and all will be there.

**Participant#4.** Wait excuse me, so if, if we want to remove something, it’s the one that is not too important.

**Participant#2.** One, order vaccines for families.

**Participant#2.** No, the vaccines.

**Participant#3.** Only sick persons.

**Participant#1.** I think, the essence of the game, we are focusing on saving the other, what are the cases, the 175 persons. Those are the persons suppose to be our main target. Because the 25 persons is already down. Because were talking about only treating the, those, those people. we were not talking about the others.

**Participant#3.** You will not treat them only. Who knows, if I go home I touch my sisters, the thing…. Yeah, the virus will… you can treat everybody.

**Participant#3.** Yea, we suppose to treat all……

**Participant#1.** I think it can be in the next option because we were thinking about reserving the others.

Recommendations

**Participant#3.** Yep, like the app Anthoinet was talking about, it will help make the work easy. Yeah, the feature of the app to be the same. And it shouldn’t, it shouldn’t be boring.

**Participant#1.** Time, for me the time is to long. It should the time should be at most after 30 minutes or maybe 15 minutes or in the soonest possible time to save life, to at least…….

**Participant#3. Number 3.** They should narrow the search options. I think it also, you don’t ever know where you going. You looking at time, you looking at money you don’t even know how to save human life. So….. the app should be like, go straight to the point.

**Participant#2.** Number 2, I think they should include more games, it makes it more interesting that give you more clear options as compare to other parts of the app

**Participant#3.** So, to conclude, the game is interesting and thanks for the time.

# Supplementary Data - Transcript from Focus Group 3

**General Feedback at Beginning of Session**

**Participant#6**. Your suppose to give us pen oo.

**Participant#4**. My first recommendation will be to the consent form. Since we are all on campus, this should be available to the students before we arrive here so we will be able to read it carefully and know exactly why we are here. It will take us 1:30 minutes to read this before taking another time to…..Yes, that’s what I’m saying, to read this why here will take time. So the next time, you have in order to give this consent form out in order to read before the student arrive here.

**Participant#4.** People signed up to that email.

**Participant#1.** People signed up to the email….. excuse me, I think to add up to that, people  signed up to the email. So it’s like, those who did not sign up to the email, did not see it. So, it’s like maybe to sign up with something for this. Like I see it, it’s totally outside of the academic… you know circle. Even thou what we are doing is somehow acedemical but it is your own availability, your own convenient, you know to be apart. So, it better to just read what you get and other things. I think most of what I saw in the email are some of the things that I expected. Even thou…..so to come up with something, you know. But the email contents are  stated here and looking at what research is like, emmm, once nobody is taking my job to carry it aroundout what you get to say how you feel about the process, so I think signing the form ………

**Participant#5**. So… excuse me sir, I just wanted to add up….. so, I’m number five right?

Yes, just to picky back on what Pap Joe said. Emmm, since indeed we signed up eemm.. for the, this meeting and…, I think it would have been proper if, or that you had a soft copy of this consent form, send it to us, we who signed up for this particular day. So we will read it, you understand….. yes because, I get to take my own time to read this stuff now and it’s going to take up time ehn you understand. So haven said that, what do I do with this? Do I write my name on it.

**Participant#2** but what your rushing us for?

**Participant#6.** Take your time.

**Participant# 3.** Number three, I want ask question. How beneficiary is this thing, Figure it out? What’s the benefit of figure it out? The benefit, the benefits it has with the students here.

**Participant#2.** Your go ahead….

**The introduction was read by participant #3 and the scenario by the lead facilitator. Feedbacks after the scenario**

**Participant#6**. Yeah, I think you will do some background, what? Number six, I think you will do some background investigation. Because you will not just act immediately. You will want to know, even thou they, they told you that they died because of some stuffs but you want to know how they prepare the food and those people that were over the food. It could be that some of them had scratches and they didn’t know. So, you will start to check back, back how everything went on before causing this problem.

**Participant#1**. Yeah, one. I, number one. Just to add up to…. You have, even thou you have money and time. But consider the background that the money you have is limited. So, you don’t want to jump into action so you don’t waste your resources, and you are not sure of what you want to do. So it’s better you do more reading so that you have the exact information, you know what you’re targeting.

**Participant#4**. Number four. While you are doing more background reading, if there’s a define channel or communication above you, they should be communicated on what is happening in this place while you are on the ground. You can stay there and do a communication, we have this incident and people have died from this wedding, while you are doing background reading.

**Participant#3.** Yeah, I want to know, is it the app that will be providing these information for you….? So, so the only thing you get to do is to maybe put in the case, or the cases then the app will carry you to those places?

**Participant#4.** Yeah number four. How will the app, take for an example I’m in Nimba, and there’s and incident in Nimba. How will the app just understand that this incidence has occurred and something needs to be done?

**Participant#6.** Decision making. Yes, decision making.

**Participant#6.** So, number six. So, I will say because from the little reading or research we did, I will say I can act because the more we try to get more information about this thing, the more other people get to die. And we don’t want people dying. Because we already know the whole base line of this whole stuff.

**Participant#5.** So, I… number five. So from the, the reading, I will think we need to act. Yeah.

**Participant#7.** Number seven. It’s not really sufficient but we can’t continue like researching when more people are dying. So for now, I think it will be important to act and then maybe later on you can continue reading.

**Participant#3.** For number three, I will say we should search. Still search.

**Participant#1.** Yeah**,** you look at the information given…..you have to look at the nature of the information. The first thing is that that information provided from our first search, its only telling you possible causes, you know that can lead to how to approach it, how to approach it, how to… what intervention to carry on, it’s not still clear yet. So, you just go further small to gain more insight of their knowledge….you know and other things. because it’s like you just spend 5 seconds on this thing. To spend another 5 seconds then one minute pass, you still get, you still get some huge time.

**Participant#3.** We can still search.

**Punching in symptoms during the first mini game**

**Participant#5.**Yeah, number five, headache

**Participant#6.** Number six, vomiting.

**Participant#1.** One. confusion, mental confusion.

**Participant#3.** Weakness.

**Participanst#5 &6.** Weakness, confusion

**Participant#5.** I will go for, I will try, I will put…. Oh, you searching ehn?

**The Exclusion part of the game**

**Participant#5.** Yes, Ebola.

**Participant#6.** I want drink water. Hajah, you get water? But I want drink, this my water here, I take it from somewhere.

**Participant#6.** Number six again, the one that was very interesting is the game part. Where you… ehnn? The, the, the search aspect, using the game to, yes.

**Participant#4**. Yea, for me…, the app gives a kind of direction trying to connect your mind to something that you need to do or think as an epidemiologist. So what really interest me is that of the situation where the case scenario gives you the possible causes.

**Participant#7.** Number seven. What really interest me is that, through the search part, where the sign and symptoms to know the multiple causes of the disease .It enable you to know how to play the game.

**Participant#4**. Yeah number four, I think the app…, it will carefully guide you from start to end.

**Participant#3.** Yea for me it was ok, I just coming across it. I really love it. The way in which went, the, excluding certain disease when it’s not part of the clinical signs and symptoms, yea.

**Participant#2**. Yea for me it was clear and well structured, and it’s very easy to work with.

**Participant#4**. From the teaching background, for me it should be more interactive. As compare to…because you facilitating you should give chance to those that also listening and or to project some of the slides and somebody from the, from the team to be able to jointly contribute and go to the next slide.

**Participant#6** Number 6 six……

**Participant#5.** Number five, what I noticed about the game, it needs, it needs  you who are using the app, if it is established you need little more education as to how to do the require, how to do the exclude or include…. Yea.. because if you don’t know like what we did first it had to tell us to go back after putting in the symptoms.

**Participant#1**. So, number one. I will say this is a step to guide to help guide people make, I think that the app needs or improve on is, is by leaving the challenge of making the decision for those using the app. Again, when case scenario is been expressed, the app should be able to say if the symptoms are listed, and it is suspected to be A,B,C,D or maybe after the suspect; or maybe we could just say like what will I do to be able to send you to another  pop up to be able to say like search for more information, and from that, after that  maybe another question to know what next? You know.. organizing resources, this and that so that it become noted. But where in somebody will say… by the time it happens, I will act and then you might go to the wrong place and get confuse and other things.

So the app should be clear and give you exact, you know? These are steps when it comes to outbreak investigation. Ehn you understand? These are just, when it comes to err, intervention but what is on hand the environment, resources available. If you get so many, for example you get A,B,C ehn you understand and you go to the next and things like that.

**Participant#5.** So, number six again, number six again. Sorry number five sir. Just to picky back on what number one said. I think the app should also try to limit the options it provides the users. So that because you talking about saving time and saving money. So, the more options you give the man, it confuses the him as to which one to take. For me, that’s how I see it.

**Participant#3.** Yea, number three**.** To also add to what he was saying, like when we came to the puzzle, it came to, to something that we have to exclude the ebola with the symptoms. So, I think the app should give the, it should go directly to the point. Like looking at malaria and ecoli. That was the two that the app that will, the app will go through before they, they explore. So what I trying to say, they shouldn’t put, they shouldn’t have put the, the ebola there. Because ebola will confuse anybody. Because if you working in time, you putting the… somebody doesn’t know ebola and you trying to discuss ebola you wasting more time, and we talking about time. so they should have just left it with only those two options..

yea illnesses that related to those symptoms, they should only be the ones that should be there.

**Would you like to play the game?**

**Participant#5.** Yeah, why not?

**Participant#3.** Number three. Yes, of course.

**Participant#7.** Even though I came in late, but from the little demonstration that was carried out for me, atleast… interesting and I enjoyed it so I will like to play it again.

**Participant#3.** Just to make it better? Number three I think you will, you will. The first button. The excluding button, and the require, ok. So I think, because you have to hit the require before going back to decide if this one… before going back. So for me, I think it should be much easier. There shouldn’t be a require button that you have to press before you go back….., you go back. I don’t know if your trying to connect what I’m saying? It should just be there soon you click on it, it should be a straight direction, soon you click it, it should just go to the search straight. If you press on weakness, it should go to the search. If you press on the one, it should go to the search. That what I trying to say. Instead of you hitting and going back to go do it again and go back. That time, it’s time consuming and time is needed to do other things.

**Participant#5.** So, you are saying

**Participant#5** number three? You can’t be hitting it one, one. According to what the app is saying. Al of the, all of those symptoms

**Participant#5.** You got to, you got to…. You can’t be hitting it and all of the, all of those symptoms that are associated with the particular scenario that are provided, you have to collect all of them, put them together then you just search..

**Participant#3.** The symptoms they are already there and they’re displaying. So all the or maybe for recommendation If the particular disease like Diarrhea, they have Diarrhea already, but you talking about if that headache it should appear in the search. Let it be like you already click on headache. Or you click on this one it should appear. You don’t have to come down and you click then you go to require then you click. Before it be appearing, that what I trying to say.

**After the first mini game (Punching in the code)**

**Participant#5.** Yea, 1, 2, 3, 4, 5

**Participant#3.** 1, 2, 3, 4, 5

**Participant#1.** 1, 2, 3, 4, 5

**Participant#5.** Test guest only.

**Participant#6.** Number six, I will go for the interview the guest.

**Participant#1.** Number one, I have a problem with those options. We are trying to solve an outbreak, so if you interview the guest, I’m confident that you will never get to the causative agent. Interview will not solve the causative agent. So, the speculation is that we are suspecting that all those that went to that party may be affected so all te causes there and the most causative agent is all there, the bacteria and all. If I interview you, you will never tell me that you have bacteria in your blood, or maybe what is causing it you know or so. So, I think those options need to be modified because it’s like, if I interview guest and the guest are together, you can’t….

Ok,.. like, like what I’m saying it’s good to solve the problem with the cause. I want to make sure that those who are ill with the symptoms and they were guest to be tested, to identify causes. Yes, to test the guest.

**Participant#4.** Yeah, number four. From the scenarios we saw, it was narrow down from the beginning for us to have an insight to what we are driving at. For that purpose, I will want an interview.

**Participant#5.** Yeah, so eh…. From the various, from the scenario provided, one would want to deduce that the scenario is concerned with food poisoning. Ehn that’s why, maybe a surveillance officer will suspect. So, if you suspect that it is food poisoning that the, the, the game, narrow down to ecoli. Emm, interviewing the guest, you have option because it also provides you an opportunity to be able to determine what kind of food was actually served at that party or at that wedding and to do an outbreak investigation.

**Participant#6.** I say interview the guest.

**Participant#1.** Just read it out for us.

**Participant#6. This participant read the results on officer focus group three**

**Participant#1.** Number one, like, already we have jump the gun. So the only thing that we can do now in the midst is.., maybe you have to or inform the authority. Because even if, like for me I’m confuse because you don’t know to say that the outbreak but even what caused the outbreak, you don’t know. Because you only interview the guest it’s like they were contacted. The interview only told you that they were contacts base on the food they ate and they were showing symptoms but what really caused the symptoms was not established. No, I will just advise authority.

**Participant#6.** Number six. I will say we will test the guest because we already know what’s happening. Other people got, some of the, they got sick. It’s possible that other people are effected but they not, they are not… the symptoms in their body are not shown to the public. So it is possible they will still have the symptoms we don’t know, so can go further to test the guest to see if whether there are other symptoms within other people before we advise the authority.

**Participant#1.** No, no, no, so we’re not testing for symptoms.

**Participant#5.** Number five. So haven seen the results from interviewing the guest, the results that are shown expose one specific symptom that is under investigation. For what I’m looking at and what testing, em what interviewing the guest shows. So I will go for testing guest.

**Are we 100% sure (confident) that we can test the guest**

**Participant#3.** Yes

**Participant#7.** I’m 100%

**Participant#5.** Princess, read for us…..

**Participant#6.** I’m tire reading oo, hahahaha

**Participant#5. This participant read the results from testing the guest**

**Participant#3. This participant read the results on officer focus group three after testing the guest.**

**Participant#1.** Yeah but whay really I think here now, you are not the final arbitrate, even thou you are the surveillance officer but the resources is from authority. So what you have the operate with per the problem that is on hand cannot cover it all. So why can’t you inform the authority because we talked about; even to test the environment.

**Participant#3.** That’s what I wanted to say.

**Mini game two (2)**

**Participant#5.** Weakness

**Participant#3.** Vomiting

**Participant#5.** Confusion

**Participant#3.** Yes, to exclude ebola

**Participant#7.** From the previous information, I belief there are things there that you will not get rid….

**Participant#1.** High fertility

**Participant#3.** That real plenty things they put there oo, require, require Liberia.

**Participant#1.** Number one. Again, what we just played by making the wrong decision is also another strategy that if this app is not improved to be a kind of a flow chat then people will get frustrated and will not want to make impart because is like you are bust with things you want help and you know you want research that will guide your path. So if the research is not giving more information and bringing you back to your question. You get frustrated and may not want to continue. Because it’s possible that they…, and are not straight forward.

**Participant#3.** The research is getting bored, yeah. Very, very boring. It na easy, going back, coming back. It’s very, very bored and that something that suppose to be like fast to get result. But you’re going coming back. Yeah.

**Participant#3.** Yeah for the..

**Participant#4.** Yes for the, number four. This was most specific then the first game.

More specific that will include the right one.

**Participant#3.** Number three. The second search I saw food born, air born, water born, but we didn’t use it.

**Participant#3.** So, I’m not saying that food born or water born. Number three I’m not saying we should put it in the search task, but then they shouldn’t be there at that time. We’re not using it at that time, so no need for them to be there.

**Participant#7. A**lvan oo,  so we choose to interview the guest, why if the answer came wrong**?**

**Participant#3.** We have to go back again because before we started this, this research is.., they gave us time and everything. We noticed that we were running…..

Ooo, okay. Wait but the money they money that the testing was charging for, that $20 what we do with it?

**Participant#2.** How many steps remaining now?

**Participant#3.** Treat only sick people who went to the wedding, option number one.

Number two,

**Participant#1.** The one that is least important, Base on the situation?

You’ll order antibiotic as the least important now because is like in your plan, people who are sick, you want to treat them. If that for them, ok. You order antibiotic I don’t know..,for the rest of the people

**Participant#2.** Those that involved…

**Participant#1.** Those that involved, because they don’t need the antibiotic.

**Participant#4.** From me, number four. The one that is least important is number one. Treat only sick people who went to the wedding. Those that also were, in the gathering, also want know their status. For me, that the best one there. To order vaccine, to order antibiotic that process. it will not be possible to other counties, it will not be possible at that time.

**Participant#1.** Yeah remove order antibiotic.

**Participant#2.** Gather more research

**Participant#1.** This game likes more research business ehn?

**Participant#7.** So, so more details thing up here oo.

**Participant#3.** Remove treat all people who went to the wedding, if even they had a particular, we are to remove right?

**Participant#7.** No, that test all people who went to the wedding to determine if they are sick.

**Participant#4.** Alvan, in most cases, number four. The outbreak is already ongoing, then you have to order vaccine from where? Taking Liberia…, ehn? When that vaccine arrives, and really made for its intended purpose, it might be late.

**Participant#7.** Number seven…..

**Participant#6.** Number six…..

**Participant#3.** Take off order vaccine for the family of the sick.

**Participant#7.** That last one there…, to help protect everybody. Vaccine across the board. Vaccine is there for prevention.

**Participant#3.** Go back again, search again

**Participant#5.** This other game own part, it will just be searching whole day?

**Participant#2.** Do the last one, just take that last one out.

**Participant#2.** But we didn’t save any life oo.

**Participant#3.** the multiple researching, yes, it was so boring and less interesting. You will go and come back again.

**Participant#6.** Number six, the, the, part that has to do with ehm, which one we were suppose to replace from the list. No, no one to remove from the list because I couldn’t identify the option that was least important for me to remove. It was boring and I got lost.

**Participant#**3.yeah, the game should be straight and to the point. It shouldn’t be giving options that are not needed at a particular time.

Yes, it should say which one to use with the correct option

**Participant#3.** Two hours.

**Participant#7.** I think the app should come with its own time but be limited.

James, but what’s the require time?

**Participant#3.** I will like the, number three. I will like this app to be something that people will just go straight and get result instead of just going. It should be concise instead of it just beating around the bush. Doing want thing to the other, wasting time to investigate. The reason for this app is to investigate, so going back and forth is very, very much time consuming. Since we sat here by 5:00 clock, if it was real something that is certain and we are sitting here to make decision, it will be problem by this time.

**Participant#3.** The search app, like the way we can go on google and get information, as fast as possible. You can compare it with any app, but I will want for it to be like search engine or google. I will like, I will recommend that the app be fast.

**Participant#1.** I will compare it to R Studio

**Participant#5.** Yea to R. Studio.

**Participant#4.** Yes, I will share my knowledge with others.

**Participant#5.** Yes, I will.

**Participant#4.** Figure it out is a.., using the app, you will not just rush in making decision is something that you must think critically. And even thou we are to be using it and give one of the most important information that will make people to like it. This app…, exactly, I will recommend it to others. Yeah.

**Participant#1.** This app could even help to know the, the outbreak response test.

**Participant#7.** Yes, I will recommend it to colleagues..

**Participant#2.** Yes, I will.
